# Diet and Systemic Lupus Erythematosus (SLE): From Supplementation to Intervention

**DOI:** 10.3390/ijerph191911895

**Published:** 2022-09-20

**Authors:** Hanxiao Jiao, Gizem Acar, George A. Robinson, Coziana Ciurtin, Elizabeth C. Jury, Anastasia Z. Kalea

**Affiliations:** 1 Division of Medicine, University College London, Rayne Building, London WC1E 6JF, UK; 2 Centre for Rheumatology Research, Division of Medicine, University College London, Rayne Building, London W1CE 6JF, UK; 3 Centre for Adolescent Rheumatology versus Arthritis, Division of Medicine, University College London, Rayne Building, London W1CE 6JF, UK; 4 Institute of Cardiovascular Science, University College London, London WC1E 6DD, UK

**Keywords:** systemic lupus erythematosus, supplementation, dietary intervention, nutrition, vitamin D, omega-3 fatty acids

## Abstract

Background: Systemic lupus erythematosus (SLE) is a chronic autoimmune inflammatory disease characterised by immune dysregulation affecting multiple organs. Current anti-inflammatory treatments used in SLE are associated with unwanted side-effects. Dietary supplementation has been suggested as a safe and effective addition to conventional treatment, but evidence of efficacy in SLE or preventing associated comorbidities is uncertain. Methods: We identified literature on clinical trials focused on nutritional interventions in SLE aiming to improve inflammation and comorbidities. A systematic-type search on Embase, Medline, and the Cochrane Library, was conducted to identify nutritional interventions among SLE patients in the past 15 years that met our inclusion criteria. Results: We identified 2754 articles, of which 14 were eligible for inclusion based on our set criteria and were subsequently quality assessed. Vitamin D or E supplementation was associated with respective improvement of inflammatory markers or antibody production, but not disease activity scores in most studies. Despite their expected synergistic actions, the addition of curcumin on vitamin D supplementation had no additional effects on disease activity or inflammatory markers. Trials of omega-3 fatty acid supplementation presented significant reductions in ESR, CRP, disease activity, inflammatory markers, and oxidative stress, and improved lipid levels and endothelial function, while a low glycaemic index (GI) diet showed evidence of reduced weight and improved fatigue in patients. Conclusions: Different dietary guidelines can therefore be implicated to target specific SLE symptoms or therapeutic side-effects. This systematic review highlights the scarcity of larger and longer in duration trials with homogenous methodologies and verifiable outcomes to assess disease progression.

## 1. Introduction

Systemic lupus erythematosus (SLE) is a chronic autoimmune disease involving both innate and adaptive immune systems [1], with increasing prevalence over the past decades [2]. SLE is characterised by circulating autoantibodies, chronic inflammation, and tissue damage affecting multiple organ systems. It is associated with comorbidities, such as cardiovascular disease (CVD) [3,4], cancer [5], metabolic syndrome, and thyroid disease [6] which affect disease symptoms and progression and increase mortality risk. Common symptoms reported by patients with SLE are fatigue and joint pain, which along with psychological manifestations such as depression, and anxiety, are affecting the quality of life and demanding the need for patient support and more effective treatment options [7].

The current management goals of SLE treatments focus on long-term survival, organ damage prevention, and life quality improvement [8] with the adverse effect of drug use as the main limitation. Chronic use of common immunosuppressive agents used in treatments for SLE, such as glucocorticoids are associated with osteoporosis and body fat redistribution, even with low-dose usage [9], while at high doses they contribute to new organ damage, such as cataracts, osteoporotic fractures, and cardiovascular damage [10]. Chronic use of immunosuppressants may also pose a heavy financial burden on patients, even in countries with well-developed healthcare systems [11].

To complement the management of SLE along with current treatments, nutritional intervention may offer a promising option. A variety of dietary components such as vitamin D, omega-3 fish oils, curcumin, glycaemic index (GI), and sodium [1,12,13] have been reported to play a role in SLE management, as described by improvements in immunological function and bone mass density. However, the way nutritional interventions and specific dietary patterns modulate immune functions in SLE and whether they can improve disease activity remains unclear. Therefore, summarising existing findings in a critical approach is essential to develop dietary interventions as a complementary treatment option.

This review aims to evaluate the effect of nutritional interventions on specific outcomes relevant to the disease progression in patients with SLE throughout the systematic review of clinical trials published over the past 15 years. Our goal was to explore the evidence base and consider any updated insights for forming dietary guidelines for this patient group.

## 2. Materials and Methods

### 2.1. Search Strategy

A systematic-type literature search was conducted to identify publications within the last 15 years (January 2006–December 2021) under the topic of this review. The reporting of this systematic review was guided by the standards of the Preferred Reporting Items for Systematic Review and Meta-Analysis (PRISMA) Statement. The three databases, Medline, Embase, and Cochrane library were searched to identify English articles using human subjects only. The searching strategy was first developed in Medline using medical subject headings (MeSHs) terms and related keywords, and the whole searching process was under the supervision of an expert librarian. We searched using the term *systemic lupus erythematosus combined with terms such as dietary supplement, diet intervention, nutrition treatment, vitamin D, vitamin E, curcumin, omega-3, fish oil, calorie restriction and glycaemic index*. Then the strategy was adapted to Embase and Cochrane separately. Detailed searching strategy and terminology can be found in Appendix A.

### 2.2. Inclusion and Exclusion Criteria

Study inclusion criteria were restricted to intervention studies and randomised controlled trials, blind or not on adult patients diagnosed with SLE, applying dietary interventions through dietary supplements or specific dietary patterns, and including a control group. We included studies testing an intervention to a group of participants followed prospectively. For the control group we defined any standard treatment, no treatment or placebo for supplementation trials or the habitual diet for dietary intervention trials. Eligible studies included a control (no-intervention) or control or comparison group (other type of intervention) and compared the effects of the intervention versus the control/comparison group. We included studies reporting the effects of the intervention on disease activity, clinical parameters, and health status of patients with SLE. Our exclusion criteria were (a) studies on experimental animal models of SLE, (b) trials which did not include a control group, (c) retrospective observational studies, (d) studies which analysed only dietary intake and serum nutrients, (e) studies which did not evaluate outcomes and other parameters relevant to disease activity.

### 2.3. Data Extraction

Two reviewers (G.A. and H.J.) screened the articles in different time points and a consensus was reached for excluded studies after discussion with a third reviewer (A.Z.K.). Firstly, studies were excluded based on title and abstract; then, full-text screening was conducted following the inclusion/exclusion criteria. Finally, critical characteristics of selected papers were extracted, including author names, publication year, country the study took place in, study design, number of patients/controls, participant characteristics, intervention characteristics and duration, and main findings reported. Studies were divided by the type of interventions to allow better comparisons among less heterogenous studies of analogous study design. Our narrative analysis and discussion addressed the potential confounding variables in each study and their impact on outcomes. To avoid bias and have a deeper understanding of the limitations of the selected studies, the quality of eligible studies was critically assessed using the Quality Criteria Checklist created by the Academic of Nutrition and Dietetics for primary research [14], based on which the studies have been classified as positive, neutral, and negative.

## 3. Results

### 3.1. Characteristics of Eligible Studies

**Figure 1:**
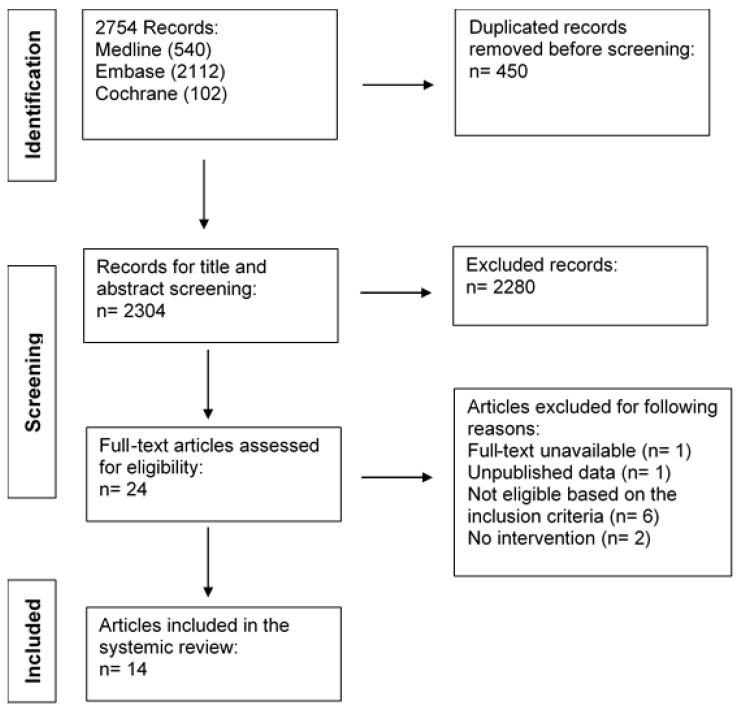
PRISMA flow diagram of the literature search in three selected databases (Medline, Embase, and Cochrane library) and the screening process of included studies.

The complete flow diagram of the screening of eligible clinical trials was created using the Preferred Reporting Items for Systematic Reviews and Meta-Analyses (PRISMA) guideline (Figure 1).

Through databases searching, 2754 records were identified. After deleting duplicated records, 2304 unique records went through title and abstract screening, and 2280 records were excluded because of ineligibility. Among those 24 articles that went through the full-text screening, one article was not accessible, one article used unpublished data not available in the paper, six studies failed to meet the inclusion criteria, and two studies did not use any intervention. Therefore, 14 articles were included in our review for further discussion and quality assessment.

The sample sizes of the 14 eligible studies ranged from 19 to 90 patients and the duration of intervention of all the included studies varied from six weeks to two years. The characteristics of these studies are displayed below, and they are divided into five tables based on their intervention types (Table 1, Table 2, Table 3, Table 4 and Table 5). Six studies [15,16,17,18,19,20] focused on the effects of vitamin D supplements among patients with SLE (Table 1), and two studies [16,20] included different analyses of the same trial but looking at different outcomes. Five articles [21,22,23,24,25] evaluated the role of omega-3 fish oils (Table 2), one trial [26] focused on the influence of vitamin E (Table 3) and one study [27] explored the effects of curcumin in the presence of vitamin D supplementation (Table 4). Only one article [28] explored the effect of low-GI diet in SLE and used as a control group a diet low in carbohydrates (Table 5). Most studies (75%) did not have restrictions on the age or sex of included participants, except for four studies [16,20,23,28] which excluded male participants, and two studies [16,20] which only included pre-menopausal women.

Ten of fourteen studies [16,17,18,20,21,22,23,25,27,28] were randomised controlled trials (RCTs). When discussing the results of the dietary interventions, most eligible studies [18,21,22,23,24,25,26,27] compared outcomes between the supplement and the placebo/control group, while some studies [15,16,17,19,20,28] reported dose-response effects. The quality assessment ratings and the characteristics of included studies are presented in Table 1, Table 2, Table 3, Table 4 and Table 5. Ten of the eligible studies [15,17,18,20,22,23,24,25,27,28] obtained a positive quality rating, indicating a low risk of bias, and the internal validity of these studies was robust for diet interventions among patients with SLE. Four studies [16,19,21,26] received a neutral rating, indicating unclear levels of validity and bias.

### 3.2. Effects of Vitamin D Supplementation Interventions in Patients with SLE

#### 3.2.1. Serum Concentration of Vitamin D

Included studies were controlled randomised intervention trials, with the exception of a prospective cross-sectional study with a dose-escalating protocol, and a prospective interventional trial (Table 1). Half of the studies [15,17,18] were limited to participants with insufficient vitamin D levels only (serum levels < 30 ng/mL, equal to 75 nmol/L) apart from Aranow et al. [17] who focused on patients with vitamin D deficiency (serum levels < 20 ng/mL, equal to 50 nmol/L). All selected trials [15,16,17,18,19,20] reported a significant increase in serum vitamin D levels in the active treatment groups. Additionally, the crossover trial by Andreoli et al. [16] reported that only an intensive dose (7.5 mg initial dose followed by 1.25 mg/month) could increase the serum vitamin D levels, while the standard dose (625 μg/month) negatively affected the vitamin D levels. Interestingly, the improvement of vitamin D levels was not sustained once the intensive supplement intake period ended. Additionally, the initial intake of vitamin D in the ‘intensive dose’ group significantly increased the serum vitamin D levels in most participants after three months. However, five patients who received calcifediol (25-hydroxyvitamin D) before joining this trial, observed a decrease in serum vitamin D after three months of supplementation. One reason can be that calcifediol might impact their vitamin D levels during the first three months, even if they stopped the intake at least one month before the entry to the trial. Finally, Aranow et al. [17] conducted a study using more intensive supplementation and compared the effect of supplements with different intake amounts. There was a slight difference in vitamin D levels between the two doses, and in the more intense group, the proportion of participants who achieved repletion of 25-hydroxyvitamin D (serum vitamin D level > 30 ng/mL) was doubled compared with the low-dose group, although the difference between the two groups was not statistically significant.

#### 3.2.2. Immune Function

Marinho et al. [19] reported an increased percentage of CD4^+^FoxP3^+^ regulatory T cells (Tregs) and decreased percentage of proinflammatory CD4^+^IL-17A^+^ T cells, which indicated an improvement in the Treg: Th17 ratio towards a more anti-inflammatory T cell profile. Similarly, Piantoni et al. [20] reported an increased percentage of peripheral-induced Tregs in both vitamin D supplement groups, and peripheral-induced increased thymic Tregs in the intensive group compared with baseline. In this study, a few participants in each group were further selected to explore cytokine production. Although interferon (IFN)-γ: IL-4 ratio reduction was not significant in patients receiving the standard dose, this ratio was reduced significantly with the intensive dose. On the contrary Aranow et al. [17] reported no significant differences in IFN-α signature response in whole blood between the placebo and supplement groups, while the expression of IFN-α-inducible genes was not correlated with serum vitamin D levels. Al-Kushi et al. [15] reported no significant improvement in immune markers in the supplementation group, as there was only slight but no significant reduction in erythrocyte sedimentation rate (ESR).

#### 3.2.3. Disease Activity

Andreoli et al. [16] did not observe a significant reduction in disease activity in any vitamin D supplementation dose, while anti-ds DNA values over the one-year interventions did not show significant changes. During the second year of the trial follow-up period, three participants experienced a disease flare, of which two patients had insufficient vitamin D levels. Another randomised, double-blinded trial conducted by Karimzadeh, Shirzadi, and Karimifar [18] with more intense supplement intake reported slight but not significant changes in the SLE disease activity index (SLEDAI) score. Aranow et al. [17] conducted a short-term trial and reported that the disease activity remained stable in supplement and placebo groups. Marinho et al. [19] provided intensive amounts of vitamin D supplements (up to 50,000 IU/week) for six months after assessing vitamin D status and adjusted their intake with updates from a three-month follow-up. This study reported a significant reduction in SLEDAI scores, although they also reported significantly decreased complement three (C3) levels. Another study by Al-Kushi [15] designed one control group and two treatment groups: one used prednisone (7.5 ± 2.3 mg/day), and the other combined the use of prednisone (7.3 ± 3.1 mg/day) with vitamin D supplement and calcium complement. The SLEDAI score, anti-ds DNA, and complement (C3 and C4) levels had a non-significant reduction in the group which was supplemented with vitamin D compared with the other groups.

#### 3.2.4. Safety

Three eligible trials [16,17,19] examined the safety of vitamin D intake in their interventions when using intensive supplement doses. In the study conducted by Marinho et al. [19], the highest dose was 1250 μg/week and was taken for a duration of eight weeks. Additionally, as mentioned before, the initial intake in the intensive supplementation group in Andreoli et al. [16] exposed participants to a substantial amount of vitamin D intake in a short period. All the interventions applied were safe among participants, including those who already had sufficient levels of serum vitamin D. Reported adverse events included three cases with slight hypercalciuria (Andreoli et al. [16]), and several mild adverse events associated with known toxicities to vitamin D including hypercalcaemia, gastrointestinal complaints, and arthralgia, and whose occurrence was balanced between placebo, low-dose, and high-dose groups (Aranow et al. [17]).

#### 3.2.5. Bone Mass Density (BMD)

Al-Kushi et al. [15] assessed BMD, providing participants a short (6 month) supplementation intervention with both cholecalciferol and calcium carbonate. Even with corticosteroid use in this group, they observed the effect of supplementation on improving BMD T-score. As expected, supplementation decreased the frequency of osteopenia from 40% to 16.7% and osteoporosis compared with baseline (26.7% vs. 13.3%), while the prevalence of osteopenia increased in both the no treatment group and the group receiving corticosteroid only. A major limitation of this study is that enrolled patients were all vitamin D deficient; therefore, these results may not be applicable to patients with normal vitamin D levels.

### 3.3. Effects of Omega-3 Fish Oil Supplementation Interventions in Patients with SLE

#### 3.3.1. Lipid Profiles and Adipokines

Four studies [22,23,24,25] analysed the changes in lipid profiles of participants. In a double-blinded RCT conducted by Bello et al. [22], the 12-week supplementation showed no effect on the low-density lipoprotein/high-density lipoprotein (LDL/HDL) ratio. However, they reported some negative outcomes in relation to cardiovascular risk, such a small average increase in total cholesterol and LDL cholesterol levels in the omega-3 group, while both biomarkers were decreased in the placebo group. In another RCT, Borges et al. [23] similarly reported increased total cholesterol and LDL cholesterol in the treatment group, and increased LDL cholesterol in the placebo group, but they all remained in normal ranges. Lozovoy et al. [24] allocated their participants into groups after stratification by demographic characteristics. They reported decreased triacylglycerol and increased total cholesterol in the omega-3 group, while no significant change occurred in the placebo group. In the double-blinded RCT conducted by Wright et al. [25], the authors reported a significant reduction in triglycerides in the omega-3 group.

Two studies [23,24] measured the changes in adipokines. Borges et al. [23] reported no significant effect of omega-3 intake in adiponectin and leptin levels. However, Lozovoy et al. [24] reported the positive effect of omega-3 in increasing serum adiponectin levels and decreasing leptin levels.

Additionally, Wright et al. [25] analysed platelet membrane fatty acids and evaluated the effects of fish oil. They observed a reduction in the percentage of arachidonic acid after the intervention in their omega-3 group. Moreover, omega-3 improved the percentage of docosahexaenoic acid (DHA) and eicosapentaenoic acid (EPA) in the platelet membrane.

#### 3.3.2. Immune Response

Four studies [21,22,23,25] assessed the effect of omega-3 supplementation on the immune responses. In the single-blinded RCT conducted by Arriens et al. [21], the treatment group was supplemented with an intensive dose (4.5 g fish oil/day for six months). When the authors compared the results between the treatment and placebo groups, and omega-3 supplementations significantly reduced ESR, indicating an improvement in systemic inflammation. They also reported an increase in the level of IL-13 and a reduction in the level of IL-12. Bello et al. [22] compared the mean change in inflammatory signals in the two groups and reported no significant difference in the levels of IL-6 and soluble intercellular adhesion molecule-1 (sICAM-1). They observed reduced soluble Vascular Cell Adhesion Molecule-1 (sVCAM-1) levels in the treatment group and increased sVCAM-1 levels in the placebo group, but the difference between these groups did not vary significantly. Borges et al. [23] reported a significant effect of omega-3 on serum C-Reactive Protein (CRP) reduction (Table 2), but no significant effect on the levels of IL-6 and IL-10. Wright et al. [25] reported slight but not significant changes in ESR and CRP levels in their treatment group after the intervention.

#### 3.3.3. Disease Activity

Disease activity was measured in four trials [21,22,24,25]. In the trial conducted by Arriens et al. [21], the Physician Global Assessment (PGA) score differed significantly between the treatment and placebo groups, indicating an improvement in PGA score. However, the SLEDAI score and renal SLEDAI score did not vary significantly between the two groups. Bello et al. [22] reported a reduction in SLEDAI score in the treatment group and increased SLEDAI score in the placebo group, but the difference between the two groups was not significantly different. The PGA score after the intervention also did not statistically differ between the two groups. Lozovoy et al. [24] reported a significant reduction in the SLEDAI score in their treatment group, suggesting reduced disease activity. This study also assessed C3 and C4 levels and anti-ds DNA titre as disease activity parameters, but these parameters did not vary significantly in the treatment group. Wright et al. [25] assessed disease activity using two different tools, the Systemic Lupus Activity Measure Revised (SLAM-R) and the British Isles Lupus Assessment Group index of disease activity for SLE (BILAG). The authors reported consistent reductions in SLAM-R scores in the treatment groups, and improvements in the joint, neuromotor, integument, and constitutional symptoms scores were observed after the intervention. They also reported consistently decreased BILAG scores, and after the intervention, significant reductions in musculoskeletal, cutaneous, cardiorespiratory, vasculitis, and general symptoms scores were observed. These results from Wright et al. [25] provide positive evidence on the benefit of omega-3 intake.

#### 3.3.4. Vascular Health

Two double-blinded RCTs [22,25] evaluated the effect of omega-3 on endothelial function. Bello et al. [22] compared the changes between the treatment and placebo groups, while Wright et al. [25] analysed “before and after” changes within the treatment and placebo groups separately. After the 12 weeks, Bello et al. [22] reported no significant difference in both brachial artery diameter results and changes in flow-mediated dilation percentage, which failed to provide evidence that omega-3 supplement intake improved endothelial functions among patients with SLE. Wright et al. [25] measured their outcomes during (12 weeks) and after the intervention (24 weeks), and flow-mediated dilatation (FMD) and diastolic shear stress significantly increased compared with baseline. At the end of the treatment, FMD was positively correlated with the percentage of DHA and EPA in platelet membranes. Wright et al. [25] also evaluated the changes in oxidative stress by analysing platelet 8-isoprostanes. They reported significant reductions in 8-isoprostanes levels in both groups, and the change in 8-isoprostanes was more extensive in the omega-3 group.

Additionally, Wright et al. [25] measured other cardiovascular parameters (systolic blood pressure, diastolic blood pressure, and heart rate), and they reported no significant difference in the treatment group, but systolic and diastolic blood pressure significantly decreased in the placebo group. The placebo group used olive oil in this study, another diet intake that can bring health benefits.

#### 3.3.5. Other Comorbidities

The trial conducted by Arriens et al. [21] assessed the quality of life in their participants. They reported that the emotional well-being in the treatment group showed an improving trend. Fatigue was measured by the energy/fatigue subscale of the RAND Short Form-36 (RAND SF-36) and Fatigue Severity Scale (FSS). The RAND SF-36 results indicated a significant trend of improvement by omega-3 intake, while the FSS scores were similar in the two groups. Notably, the RAND SF-36 results in the treatment group at baseline were significantly worse than the placebo group in many aspects, including fatigue and emotional well-being. This difference should be considered when evaluating the effect of omega-3 on fatigue.

#### 3.3.6. Safety

No severe adverse events were reported in eligible studies. Arriens et al. [21] reported that gastrointestinal side effects in seven participants resulted in withdrawal from the study, but the rate of these events between groups was not significantly different. Bello et al. [22] reported six adverse events unrelated to omega-3 intake, and there was no patient withdrawal in their study. In the treatment group from Borges et al. [23], one participant experienced diarrhoea and another experienced fish aftertaste. These findings indicate that omega-3 supplements are well-tolerated in patients with SLE.

### 3.4. Effects of Vitamin E Supplementation Interventions in Patients with SLE

Maeshima et al. [26] selected participants with Raynaud’s phenomenon or fingertip ulcers into the vitamin E treatment group (Table 3). The authors assessed oxidative DNA damage by urinary 8-hydroxydeoxyguanosine (8-OHdG) and regarded anti-ds DNA titre as a disease activity predicting parameter. The assessment was conducted in two time points taking into consideration that exposure to sunlight is a well-established environmental factor which induces or exacerbates symptoms of SLE. They reported that the amount of daily prednisolone dose was similar in the two groups. They observed reduced anti-ds DNA titre in the intervention group, which was supplemented with vitamin E, suggesting a role of vitamin E in regulating antibody production, independent of its antioxidant role. The urinary 8-OHdG levels did not vary significantly in the two groups.

The limitation of this study is the fact that a lot of essential information on the study design is missing such as the exact doses of vitamin E supplements and other essential data on the methodology, factors that affect its validity. The amount of vitamin E intake and the intervention duration were described using ranges and the sample selection was not free from bias. Additionally, demographics only contained age and SLEDAI score, which were insufficient to decide whether these groups were comparable.

### 3.5. Effects of Curcumin Supplementation Interventions on Patients with SLE

The double-blinded RCT conducted by Singgih Wahono et al. [27] provided both groups with vitamin D supplements, and the intervention group received *Curcuma xanthorrhiza* in addition to this (Table 4). They reported significantly higher transforming growth factor (TGF)-𝛽1: IL-6 ratios in the intervention group. However, the improvements in serum levels of vitamin D and cytokines (increased TGF-𝛽1, decreased IL-6) and reduced disease activity did not vary between the groups. They also reported a positive but moderate correlation between the reduction in IL-6 levels and the reduction in SLEDAI in all those participants with insufficient vitamin D levels. Additionally, a quarter of participants experienced vitamin D decline in the intervention group, and 21% of this group suffered reductions in TGF-𝛽1 levels. It should be noticed that the outcomes in the intervention group were the overall effect of vitamin D and curcumin, which did not represent the effect of curcumin intake in SLE.

### 3.6. Effects of Dietary Restriction Interventions on Patients with SLE

The RCT conducted by Davies et al. [28] selected SLE patients who received corticosteroids and were overweight at baseline and divided them into a low-GI dietary intervention group or a calorie-restricted intervention group (control group). The two restrictive patterns were well-tolerated by participants. The authors reported significant reductions in weight, waist, and hip measurements in both groups (Table 5). The changes in the parameters above did not vary significantly between groups. For those participants with fatigue, diet restrictions significantly reduced their FSS scores in both groups. The authors measured disease activity by SLEDAI, BILAG, the European Community Lupus Activity Measure (ECLAM), and Systemic Lupus International Collaborating Clinics/American College of Rheumatology (SLICC/ACR) damage scores but did not observe significant improvement in disease activity in either group. Moreover, neither group observed the effects of diet restrictions on sleep quality and cardiovascular parameters.

## 4. Discussion

### 4.1. Vitamin D and SLE

Vitamin D is often considered as an anti-inflammatory agent. Previous studies have suggested the potential role of vitamin D in regulating both innate and adaptive immunity, as immune cells express vitamin D receptor, including antigen-presenting cells, T cells and B cells [29]. Recent studies have proved that vitamin D intake can decrease inflammatory cytokines, suppress disease progression, and increase Tregs in mouse models with SLE [30]. Additionally, one in vitro study reported that vitamin D exposure reduced apoptosis and modified cell cycle progression and the expression of apoptotic genes in samples isolated from patients with SLE [31].

The primary source of vitamin D is obtained through ultraviolet light exposure [32]; vitamin D can also be obtained at a limited amount from foods including fortified dairy and fatty fish [33]. Evidence has shown that compared with the general population, vitamin D deficiency is more frequent in patients with SLE, partly because patients are advised to avoid sunlight to prevent flares; chronic renal disease and the use of glucocorticoids can also affect the level of vitamin D [34]. Additionally, some studies reported the relationship between vitamin D deficiency and enhanced disease activity of SLE, suggesting the role of low vitamin D levels in disease progression in SLE [35]. Thus, vitamin D supplements are considered beneficial among patients, and vitamin D_3_ is preferred over vitamin D_2_ because it is more efficient in improving the serum vitamin D levels and has longer shelf life [34].

This review highlighted the effect of vitamin D in modulating Tregs and Th17 cells; similarly, another single group study also reported decreased Th1 and Th17 cells and increased Tregs [36]. One included study [17] failed to observe the regulatory effect of vitamin D on IFN-signature. This failure may be due to the limited numbers of IFN-inducible genes analysed; also, the duration of the intervention was short compared with other studies. Additionally, in SLE, not all patients exhibit an IFN gene signature [37], so the included study outcomes do not mean that vitamin D supplementations have no effect on immune responses.

Although several trials reported regulations in immunological response, the improvement in disease activity has not been vigorously observed, which can be partly explained by the heterogeneity of SLE mechanistic pathogenesis and presentation. Notably, many trials selected patients with stable and not active disease activity to avoid flare during the intervention, which made observing the changes in disease activity more difficult. One trial [19] provided supplements based on patients’ vitamin D levels, which was the only study that significantly reduced the SLEDAI score, although this study also reported negative C3 outcomes, which suggested more active disease activity. The improvement in the SLEDAI score suggests a potential improvement in disease activity and may be explained by the optimised benefit from personal vitamin D intervention. However, the outcomes from this study require further exploration in disease activity, especially the C3 levels.

According to one included study [15], vitamin D and calcium supplementations improve BMD and reduce the frequency of osteopenia and osteoporosis even under receiving corticosteroids. Another study also reported the association between BMD reduction and lack of vitamin D supplements in females with juvenile SLE [38]. Thus, vitamin D supplements are critical in protecting patients from SLE disease-associated damage and the adverse bone events of traditional treatment.

General international recommendations suggest vitamin D complementary intake of 20–25 μg/day, or 1250 μg/month, is safe for most individuals [1]. However, this review provides different suggestions for patients with SLE. Most selected trials showed that intensive doses were well-tolerated, indicating vitamin D supplements up to 1250 μg/week and 7.5 mg bolus for only a single intake was safe. Moreover, vitamin D dose at 625 μg/month might even negatively affect the level of vitamin D in patients with SLE. Furthermore, it seems that within the safe range, more intensive supplement intake is more effective in restoring vitamin D levels, allowing more patients with vitamin D deficiency to achieve repletion of serum vitamin D. Lastly, to avoid toxicity by excessive vitamin D intake, personal treatment plans and regular follow-up adjustment should be considered.

### 4.2. Omega-3 and SLE

Omega-3 fatty acids have anti-inflammatory effects by regulating the level of inflammatory mediators and CRP [13]. Among omega-3 polyunsaturated fatty acids (PUFAs), DHA and EPA are the most biologically active, able to regulate pro-inflammatory cytokine production, cytotoxic activity mediated by T cells, and macrophages and neutrophil/monocyte chemotaxis [1]. However, patients with SLE are characterised by altered lipid profiles and lowered omega-3 PUFAs levels, which might relate to an increased frequency of cardiovascular complications [39]. Therefore, dietary omega-3 supplements can be helpful; researchers have reported reduced levels of inflammatory markers and autoantibodies as well as improved lifespan in mouse modules with SLE [40]. Previous positive clinical outcomes also prove the benefit of omega-3 PUFAs supplements in other autoimmune diseases, such as rheumatoid arthritis [41].

Included studies have shown that omega-3 PUFAs intake can decrease serum triacylglycerol, and they can also improve platelet membrane fatty acids by decreasing the percentage arachidonic acid while increasing the percentage DHA and EPA. In addition, omega-3 PUFAs intake may also increase serum levels of adiponectin and reduce leptin levels. However, increased total cholesterol and LDL cholesterol levels are also commonly observed among included studies. This is a critical finding, since the occurrence of dyslipidaemia, CVD, and cerebrovascular disease in patients with SLE is elevated compared with the general population [6]. Future RCTs with larger sample sizes, longer duration, and extended lipid profile parameters are required to further explore the effect of omega-3 intakes on lipid profiles and adipokines, as well as the negative impacts of omega-3 on lipid profiles.

Only one selected trial [21] reported significant improvements in inflammatory cytokines and systemic inflammation. Other trials reported differences in cytokines or ESR that were not statistically significant. Although some trials regarded CRP levels as an indicator of inflammation, evidence shows that CRP is not sensitive in SLE and is not an effective marker for most patients [42,43].

Different from the outcomes in inflammatory markers, most trials reported the effect of omega-3 intake on improving disease activity under several measurements, including PGA, SLEDAI, SLAM-R, and BILAG. The involvement of multiple systems in SLE can partly explain this as the improvement of disease activity can relate to other aspects instead of inflammation, such as joint, neuromotor, cutaneous, and vasculitis aspects. Meanwhile, different methods might present different results as they are measured in different ways. Therefore, using two scales is effective to avoid missing any improvements. For example, when measuring SLE disease activity, one advantage of PGA is that this measurement is not limited by predefined manifestations or organ systems, which allows PGA to capture changes in all the heterogeneous aspects of disease activity [44].

Aside from disease activity, many studies report the presence of accelerated atherosclerosis among patients with SLE and its impact on mortality [45], and atherosclerosis is characterised by endothelial dysfunction [46]. A typical measurement for evaluating endothelial function is FMD [12]. Additionally, assessing oxidative stress is another measure. Oxidative stress can directly affect vascular tone, which can alter nitric oxide bioavailability and signalling, resulting in endothelial dysfunction [46]. According to one included study [27], omega-3 PUFAs intakes improved endothelial function and reduced oxidative stress; the correlation of percentage DHA and EPA in platelet membrane and FMD also suggested the benefit of improved lipid profiles by omega-3 intake. Still, this trial failed to observe the effect of omega-3 on cardiovascular parameters. These outcomes suggest the potential role of omega-3 PUFAs in improving endothelial function, and they require further trials to test the reliability in a larger sample of patients with SLE.

Only one interventional trial [21] evaluated the impact of omega-3 on fatigue and emotional well-being. Even though there was a trend of symptoms improvement, this did not reach statistical significance perhaps due to the fact that this study was underpowered for the fatigue and quality of life outcomes. A previous meta-analysis exploring the impact of omega-3 intake on depressive disorders also show improvements in patients with depression or depressive symptomatology [47]. Considering the frequency of fatigue and depression among patients with SLE, omega-3 supplements can be critical in improving patients’ quality of life.

In clinical practice, several reasons might explain the non-significant outcomes. For example, a recent meta-analysis suggests that patients with more active SLE disease activity at baseline tend to have more pronounced changes after omega-3 treatments [40]. This study also believes that patients with autoimmune diseases may benefit more from omega-3 supplementation if they had low fish consumption before. Still, unlike other eligible trials, Bello et al. [22] failed to observe any improvements in lipid profiles, inflammatory parameters, disease activity measurements, and endothelial function; even the authors could not explain the possible reasons, leaving confusion and making the outcomes less convincing.

When considering dose usage for future studies, it should be noticed that high doses may negatively disturb the necessary inflammation during infection or result in potential cardiovascular effects [48]. In this review, most selected trials used a low dose (3 g/day); a dose up to 4.5 g/day can be well-tolerated among patients. Another review also suggested that the relative amounts of DHA and EPA can be critical as DHA and EPA do not work collaboratively on improving all aspects of vascular functions [12].

### 4.3. Vitamin E and SLE

Vitamin E is known for its antioxidant function, suggesting a potential regulatory effect in the immune system by stimulating protective mechanisms [49]. A previous study in a mouse model of SLE reported that vitamin E supplementation decreased oxidative stress, secretion of pro-inflammatory cytokines, and expression of Major Histocompatibility Complex (MHC) class II, while the vitamin E-supplemented diet also changed the composition of splenocyte fatty acid [50]. However, the effect of vitamin E is controversial. In mouse models, research has previously reported that high vitamin E intake inhibits Th1 response, which might fail to benefit Th2 prone autoimmune diseases, including SLE [51].

According to the findings of the study included in this review [26], vitamin E can regulate antibody production and suppress autoantibody production among patients with SLE, an effect independent of its antioxidant role; the effect on markers of disease activity was not significant. Incomplete information in the study design limits further analysis and discussion of this preliminary study, while no other trials investigated vitamin E as an intervention. Thus, despite the interesting hypothesis, the evidence is inconclusive on the effect of vitamin E in patients with SLE, and further studies are required with larger sample sizes and better study design such as a clear rationale on the supplementation dose of the vitamin E dose, given the effects on high-dose vitamin E supplementation on increasing all-cause mortality in patients with chronic disease [52].

### 4.4. Curcumin and SLE

Curcumin is a polyphenol compound of turmeric with anti-inflammatory and antioxidant activities [53]. Few trials have been conducted in SLE or in the subset of patients who develop lupus nephritis (LN). The frequency of LN ranges from 40 to 70% among patients with SLE and is responsible for the high morbidity and mortality rate of the disease [54]. A previous study reported that curcumin intake can protect mouse models from LN by reducing renal damage, regulating pro-inflammatory cytokines and antibody production [55]. One in vitro trial reported improvement in the proliferation of peripheral blood mononuclear cells in LN patients [56], and another observed the modulatory effect of curcumin on Th17/Treg balance on CD4^+^ T cells in patients with SLE [57]. One RCT conducted in SLE reported that short-term curcumin supplementation was safe and can benefit patients who suffer from LN by decreasing proteinuria, haematuria, and systolic blood pressure [58]. Thus, evidence suggests a role of curcumin in improving inflammation and renal condition among patients with SLE.

The included trial [27] evaluated the synergistic effect of curcumin combined with vitamin D instead of the effect of curcumin in patients with SLE. Except for the higher TGF-*β*1/IL-6 ratios in the intervention group, the added curcumin did not improve the overall treatment outcome compared with the placebo group. One potential explanation can be the low dose of curcumin, as *Curcuma xanthorrhiza* in the above study only contains around 1–2% of the active compound curcumin [27]. Moreover, the bioavailability of curcumin is low, which might affect the intake efficiency [53]. In the absence of adequately supported safe dose range for supplementation, curcumin dose usage requires caution, as several negative effects in mouse models have been reported, including altered central nervous system and brain atrophy [1]. In addition, curcumin intake in this study seemed to affect the capability of vitamin D supplementation in some respects, which was attributed to the capacity and efficacy of both curcumin and vitamin D to bind to vitamin D receptors [27]. Thus, applying these two supplements together may not be a the most advisable strategy.

### 4.5. Diet Restrictions and SLE

Some trials combine the influence of several dietary intakes and apply diet restrictions in patients with SLE. Recent reviews already suggest some beneficial effects of controlling macronutrients intakes, such as protein and calorie intake restriction [1,13]. The frequency of obesity is elevated in SLE [6], likely associated with the effect of corticosteroid usage. The selected RCT [28] demonstrates that both a low-GI diet and a calorie-restricted diet can reduce weight and improve fatigue in patients with SLE, which is beneficial for their health and quality of life. Still, this RCT has limitations; for example, the small sample size of this trial might limit the effect of diet restriction, such as the assumed cardiovascular benefit of the calorie-restricted diet [28].

One recent study reported the effect of a short-term sodium restriction diet in autoimmune disease [59]. The authors observed reductions in the percentage of Th17 cells and IL-9 levels and increased percentage of Tregs in a group of SLE patients, indicating an improvement in pro-inflammatory response. This evidence suggests a new direction for diet restrictions in adaptive immunity, and future studies at larger scales can explore that further in SLE. Additionally, a Mediterranean-style diet is suggested to be beneficial in SLE as its compounds have anti-inflammatory properties [60]. Still, no clinical evidence of this diet in SLE is available, and future exploration in clinical experiments are required.

### 4.6. Implications for Future Research

Plenty of opportunities lie in the direction of nutritional interventions for future exploration. For example, when considering using vitamin D and omega-3 as treatments for patients, future clinical trials should achieve larger sample sizes and durations, as well as expanded testing parameters and ranges (e.g., more comprehensive examination of immune cells responses, cytokine profiles, lipid profiles, and gene expression signatures) to understand their protective mechanism in SLE better. Meanwhile, the intensity of the intervention needs more studies to provide a safe and effective range to guide doctors and patients with SLE. Engaging the patients from the early steps of study design should also be a priority, to improve adherence to the intervention [61]. For vitamin E and curcumin, before conducting trials in patients, more studies using animal models or in vitro trials are necessary to test their anti-inflammatory or antioxidant properties with safety ensured. In addition, several dietary patterns mentioned require trials to be conducted with appropriate patient populations, larger sizes, and longer duration to explore their effects while avoiding adverse events. Our recent survey reported that SLE patient populations are very keen to explore a range of dietary modifications aiming to alleviate symptom severity [62]. Thus, it is important to conduct clinical trials on the effects of dietary interventions and to provide the evidence-based information required to inform patient choices and guide healthcare providers to improve the health and quality of life for patients with SLE.

### 4.7. Limitations

This review contains a broad systematic literature search covering well-designed human trials in the past 15 years, with criteria-based selection, quality assessments, comprehensive referencing, and critical discussion. However, there are some limitations. Data about efficacy of various nutritional interventions in SLE are limited, and some of the studies included in this systematic review reported inadequate information. Additionally, most eligible trials were conducted with small size study groups and had short duration follow-ups. All these factors can affect the observation of potential effects or limit the effect size of the evidence. Moreover, some studies used different methods measuring the same clinical parameter, and some used different statistical methods, making the comparison between them less accurate. The validity of a few of the outcome measures is hard to be assessed, and this becomes an important limitation especially in studies which report variation in outcomes. Lastly, in some cases, only one eligible human study was conducted, making the evidence inconclusive. Assessing the study quality using the Quality Criteria Checklist was useful in confirming the quality of evidence provided by a longer term well-designed RCT over a preliminary trial that aims to assess either the efficacy or effectiveness of an intervention. Thus, some of the preliminary or pilot studies included in our review provide promising effects that should be interpreted with caution.

## 5. Conclusions

Based on available evidence, the present systematic review reveals that vitamin D supplementation can increase its serum levels, reduce inflammation, and may benefit disease activity and bone health. Omega-3 supplementation lowers disease activity and may reduce inflammation and oxidative stress, improve lipid profiles and endothelial function, and even help to improve the quality of life. Vitamin E supplementation may regulate antibody production. A low-GI diet can aid weight loss and reduce fatigue in patients. The synergistic effect of curcumin and vitamin D is not more efficient than vitamin D supplementation, suggesting that separate supplementations may achieve better outcomes. All the doses used in various trials were well-tolerated and this evidence can be used as safety references for future studies. Further investigations with more extensive trials and better methodological quality are required to examine the validity of this systematic review findings and explore other areas of research such as long-term benefits for disease control in SLE, as well as impact on comorbidities and quality of life overall.

## Figures and Tables

**Table 1:** Characteristics of included studies on vitamin D supplementation interventions.

Study (Country)	Study Design	Sample Size (n)	Intervention *(Control Group)*	Duration (Year (yr); Month (m); Week (wk))	Main Findings	Quality Assessment Rating
Andreoli et al., 2015 [16] (Italy)	Randomised prospective study with cross-over design	34 female patients with SLE	Vitamin D_3_ supplements; switch to the alternative dose after one year Intervention: Intensive Dose: 7500 μg vit D_3_ initial intake, then 1250 μg/m as maintenance *Control Group: Standard Dose: 625 μg/m*	2 yr	Intensive vit D supplement dose was safe and restored vit D (higher ratio of patients in the sufficiency range when compared with the same ratio in control group (75% vs. 28%, *p*.0.001). Control group: negative effect on 25− OH vit D levels, with a decline in the rate of sufficient patients from 64% to 38%. No significant changes in disease outcomes. Possible selection bias.	Ø
Piantoni et al., 2015 [20] (Italy)	Randomised prospective study with cross-over design	34 female patients with SLE	Vitamin D_3_ supplements for vit D deficient patients; switch to the alternative dose after one year Intervention: Intensive Dose: 7500 μg vit D_3_ initial intake, then 1250 μg/m as maintenance *Control Group: Standard Dose: 625 μg/m*	2 yr	Enhanced regulation T cells, seemed to have immunomodulatory effect. Intervention group: reduction in the IFN-γ/IL-4 ratio (from 12.1 to 3.2; *p* = 0.01) among CD8+T cells. The reduction in this ratio in the control group was not statistically significant (from 7.5 to 5.6) suggesting a role of vit D in modulating cytokines balance: supressed Th1 pathway and promoted Th2 pathway.	+
Aranow et al., 2015 [17] (USA)	Double-blind placebo-controlled trial	3 male, 51 female patients with SLE	Interventions: Low-dose group: oral vitamin D_3_ 50 μg/d High-dose group: 100 μg/d *Control Group: Placebo*	12 wks	Vit D supplementation restored vit D levels. High-dose supplementation was safe. No changes in expression of IFN signature and IFNα-inducible genes. Disease activity observed in both groups remained stable and was independent of supplements.	+
Shirzadi, Karimzadeh and Karimifar, 2017 [18] (Iran)	Double-blind placebo-controlled RCT	9 male, 81 female patients with SLE	Intervention group: Oral vitamin D_3_ 1250 μg/wk for first 3 m, then 1250 μg/m for 6 m *Control Group: Placebo*	9 m	Vit D supplements significantly improved vit D levels in intervention group (17.36 ± 4.26 ng/mL vs. baseline 37.69 ± 5.92 ng/mL, *p* < 0.001). The mean of vitamin D had no significant difference before and after intervention in placebo group (16.78 ± 4.39 ng/mL vs. 16.62 ± 4.61 ng/mL, *p* = 0.53). No significant improvement in disease activity (mean of disease activity (SLEDAI) was not different significantly before and after vit D administration (3.09 ± 2.36 vs.1.62 ± 1.25, *p* = 0.39).	+
Marinho et al. 2017 [19] (Portugal)	Prospective cross-sectional study with dose escalating protocol	1 male, 23 female patients with SLE	Intervention dose was determined based on patients’ vit D levels *Baseline:* <50 nmol/L: 1250 μg/wk cholecalciferol/ for 8 wks, then 50 μg/d >50 nmol/L and <75 nmol/L: 100 μg/d for 8 weeks, then 50 μg/d >75 nmol/L: 50 μg/d *3-month follow-up:* <50 nmol/L: 1250 μg/wk cholecalciferol/for 8 wks, then 50 μg/d >50 nmol/L and <75 nmol/L: 100 μg/d for 8 weeks, then 100 μg/d >75 nmol/L and <125 nmol/L: 50 μg/d >125 nmol/L: 25 μg/d	6 m	Vit D supplementation -safe therapy; significantly increased vit D levels; decreased disease activity; beneficial immunological effects: increased FoxP3^+^ expression in CD4^+^ T cells, decreased CD4^+^IL-17A, improved Treg/Th17 ratio, an effect described for the first time in SLE patients, of real benefit, as shown by the effective decrease in the SLEDAI scores. Highlighted the importance of individualised supplements dose for patients.	Ø
Al-Kushi et al., 2018 [15] (Saudi Arabia)	Prospective interventional study	15 male, 66 female patients with SLE	Intervention: Corticosteroid Treatment w/Supplementation Group: Mean prednisone dose: 7.3 ± 3.1 mg/d, with (35 μg cholecalciferol + 1250 mg calcium carbonate tablet/d) *Control groups:* *No Corticosteroid Treatment Group* *Corticosteroid Treatment Only Group: mean prednisone dose: 7.5 ± 2.3 mg/d*	6 m	Vitamin D and calcium intake benefited the side effect of corticosteroids. Significantly increased serum vitamin level. Significantly increased bone mass density and decreased the frequency of osteopenia and osteoporosis. Intervention group: BMD improvements in T-scores (*p* = 0.002); the frequency of osteopenia decreased from 40% (n = 12) at baseline to 16.7% (n = 5); frequency of osteoporosis decreased from 26.7% (n = 8) to 13.3% (n = 4). In the other two control groups after 6 months osteopenia prevalence increased while there was no change in the number of osteoporotic patients. No significant improvement in immune markers and disease activity.	+

IFN: interferon; IL: interleukin; RCT: randomized controlled trial; USA: the United States of America; Quality Assessment Rating: +: positive; Ø: neutral.

**Table 2:** Characteristics of included studies on omega-3 supplementation interventions.

Study (Country)	Study Design	Sample Size (n)	Intervention *(Control Group)*	Duration	Main Findings	Quality Assessment Rating
Arriens et al., 2015 [21] (USA)	Single-blind (patients) placebo-controlled RCT	7 male, 25 female patients with SLE	Intervention group: 6 capsules fish oil/day (2.25 g EPA and 2.25 g DHA) *Control group:* 6 capsules placebo/day (purified olive oil)	6 m	Fish oil intake resulted in a trend of improvement in fatigue and emotional well-being under the RAND SF-36 scale in treatment (median change of 10) vs. placebo (−2.50), *p* = 0.092, but no significant difference in FSS score. Improved disease activity under PGA score in treatment (median change of −0.550) vs. placebo (0.50), *p* = 0.015, but no significant change in SLEDAI scores. Significantly decreased ESR in treatment (median change of −5.0 mm/h) vs. placebo (4.5 mm/h), *p* = 0.008 and IL-12 levels (−16.13 vs. 8.54, *p* = 0.058), but increased IL-13 levels (−3.89 vs. −16.86, *p* = 0.033), suggesting reduced inflammation.	Ø
Bello et al., 2013 [22] (USA)	Double-blind placebo-controlled RCT	5 male, 80 female patients with SLE	Intervention group: Omega-3 3 g (1.8 g EPA and 1.2 g DHA) *Control group:* Placebo (corn starch)	12 wks	Omega-3 intake might increase total cholesterol and LDL-cholesterol (average increase in treatment group of 3.11 ± 21.99 mg/dL vs. placebo of 1.87 ± 18.29 mg/dL, *p* = 0.0266). No significant differences in flow-mediated dilation, inflammatory markers, and disease activity in the two groups.	+
Borges et al., 2017 [23] (Brazil)	RCT	49 female patients with SLE	Intervention group: Oral omega-3 fatty acids (1.08 g EPA and 0.2 g DHA/d) *Control group:* Maintain habitual diet	12 wks	Omega-3 supplements decreased serum CRP level in the treatment group (median from 5.0 (4.9–8.1) to 4.9 (4.9–7.2) ), compared with an increase in the control group, *p* = 0.008). No significant differences in IL-6 and IL-10 cytokines, adiponectin, and leptin levels. Omega-3 intakes increased serum cholesterol (median from 168.0 (151.0–194.0) to 188.0 (162.0–214.5), *p* = 0.12) and LDL-cholesterol (median from 95.0 (80.0–116.0) to 115.5 (90.0–129.2), *p* = 0.003), although they remained within normal limits.	+
Wright et al., 2008 [25] (UK-Northern Ireland)	Randomised intervention trial	4 male, 56 female patients with SLE (4 dropped out)	Intervention group: Omacor 4 capsules/d (1.8 g EPA and 1.2 g DHA) *Control group:* Placebo 4 capsules/day (olive oil)	24 wks	Low-dose omega-3 PUFAs supplement improved disease activity; SLAM-R (from 9.4 (SD 3.0) to 6.3 (2.5), *p* < 0.001) and BILAG (from 13.6 (6.0) to 6.7 (3.8), *p* < 0.001). It also improved endothelial function; reduced FMD (from 3.0% (−0.5 to 8.2) to 8.9% (1.3 to 16.9), *p* < 0.001). It also reduced oxidative stress; platelet 8-isoprostanes (from 177 pg/mg protein (23–387) to 90 pg/mg protein (32–182), *p* = 0.007).	+
Lozovoy et al., 2015 [24] (Brazil)	Double-blind placebo-controlled trial	5 male, 57 female patients with SLE	Intervention group: Fish oil n-3 fatty acids 3 g/d (10 capsules, equal to 1.8 g EPA and 1.2 g DHA originated from sardines) *Control group:* Maintain habitual diet	4 m	Fish oil supplement significantly decreased triacylglycerol (from 112.0 (69.0–143.0) to 95.5 (79.3–129.8), *p* = 0.039) but increased total cholesterol (from 193.0 (162.0–216.0) to 205.0 (181.8–232.3), *p* = 0.026). Observed increased plasma adiponectin (*p* ˂ 0.026) and reduced leptin (*p* ˂ 0.024) level, suggesting potential benefit of reducing cardiovascular risk. Also observed a significant reduction in disease activity (from 2 (0–10) to 0 (0–6), *p* = 0.0232) in the treatment group.	+

CRP: C-reactive protein; DHA: docosahexaenoic acid; EPA: eicosapentaenoic acid; ESR: erythrocyte sedimentation rate; FSS: fatigue severity scale; IL: interleukin; LDL: low-density lipoprotein; PGA: physician global assessment; PUFAs: polyunsaturated fatty acids; RAND SF-36: RAND Short Form-36; RCT: randomized controlled trial; SLE: systemic lupus erythematosus; SLEDAI: systemic lupus erythematosus disease activity index; UK: the United Kingdom; USA: the United States of America; Quality Assessment Rating: +: positive; Ø: neutral.

**Table 3:** Characteristics of included studies on vitamin E supplementation interventions.

Study (Country)	Study Design	Sample Size (n)	Intervention	Duration	Main Findings	Quality Assessment Rating
Maeshima et al., 2007 [26] (Japan)	Non-randomised intervention trial (Preliminary study)	3 male, 33 female patients with SLE	Intervention group: Oral vit E 150–300 mg/d with PSL *Control group:* PSL only	3 to 48 months (22.8 ± 16.8 months)	Lower anti-ds DNA antibody titre in the intervention group vs. treatment group (during intense sunlight: 17.9 ± 20.3 IU/L vs. 66.3 ± 76.8 IU/L, respectively; during the remainder of the year 16.3 ± 19.4 IU/L vs. 55.8 ± 59 IU/L, respectively) No significant difference of urinary 8-OHdG observed. Vitamin E might regulate antibody and autoantibody production independent of antioxidant activity.	Ø

Anti-ds DNA: anti-double stranded DNA; 8-OHdG: 8-hydroxydeoxyguanosine; PSL: prednisolone; Quality Assessment Rating: Ø: neutral.

**Table 4:** Characteristics of included studies on curcumin supplementation interventions.

Study (Country)	Study Design	Sample Size (n)	Intervention	Duration	Main Findings	Quality Assessment Rating
Singgih Wahono et al., 2017 [27] (Indonesia)	Double-blind RCT	39 patients with SLE	Intervention group: Cholecalciferol 30 μg/d + *Curcuma xanthorrhiza* 60 mg/d *Control group:* Cholecalciferol 30 μg/d + placebo tablet/d	3 m	Increased serum vitamin D, TGF-𝛽1 level, decreased IL-6 level and improved disease activity in both groups. No different effect by curcumin supplements. SLEDAI score had a moderate positive correlation with serum IL-6 level (r = 0.569, *p* = 0.000) but not with TGF-β1 (r = 0.055, *p* = 0.74).	+

IL: interleukin; SLEDAI: systemic lupus erythematosus disease activity index; TGF: transforming growth factor; Quality Assessment Rating: +: positive.

**Table 5:** Characteristics of included studies on the effect of specific dietary patterns.

Study	Study Design	Sample Size (n)	Intervention	Duration	Main Findings	Quality Assessment Rating
Davies et al., 2012 [28] (UK)	Clinical trial	23 female patients with SLE	*Low-GI diet*: Carbohydrate 45 g/d with low-GI food, no restriction of protein and fat *Macronutrient composition (% total energy intake*, *K*cal/day): 10–15% carbohydrate, 25% protein, 60% fat (saturated and unsaturated); calories *ad libitum* *Low-calorie diet:* 2000 Kcal/day *Macronutrient composition (% total energy intake):* 50% Carbohydrate, 15% Protein, 30% Fat *Study Duration:* 6 weeks	6 wks	Both low-GI and low-calorie diets were safe and well-tolerated. Significant weight loss in both groups of participants who use low-dose corticosteroid: low-GI diet group 3.9 ± 0.9 kg; low-calorie diet group 2.4 ± 2.2 kg; *p* < 0.01 for both groups from baseline values. No significant difference in the effect of two diets. No significant changes within or between groups in disease activity, cardiovascular biomarkers, insulin sensitivity. Weight loss and diet intervention might contribute to improvement in fatigue.	+

GI: glycaemic index; UK: the United Kingdom; Quality Assessment Rating: +: positive.

## Appendix A

The searching strategy on each database is displayed below.

Database: Ovid MEDLINE(R) ALL <1946 to 22 June 2021>

Date: 23 June 2021

Search Strategy:

--------------------------------------------------------------------------------

1   Lupus Erythematosus, Systemic/(56,217);

2   Systemic lupus erythematosus.mp. [mp = title, abstract, original title, name of substance word, subject heading word, floating sub-heading word, keyword heading word, organism supplementary concept word, protocol supplementary concept word, rare disease supplementary concept word, unique identifier, synonyms] (53,184);

3   1 or 2 (72,487);

4   Dietary Supplements/(62,944);

5   Exp vitamin D/(62,021);

6   Vitamin E/(26,996);

7   Curcumin/(11,726);

8   Exp Fatty Acids, Omega-3/(26,131);

9   Diet/or diet, carbohydrate-restricted/or diet, fat-restricted/or diet, Mediterranean/or diet, protein-restricted/or diet, reducing/or diet, sodium-restricted/or caloric restriction/or recommended dietary allowances/or nutritional status/(238,567);

10   Diet, Healthy/(5092);

11   Dietary supplements.mp. [mp = title, abstract, original title, name of substance word, subject heading word, floating sub-heading word, keyword heading word, organism supplementary concept word, protocol supplementary concept word, rare disease supplementary concept word, unique identifier, synonyms] (67,715);

12   (Diet* adj3 intervention*).tw. (13,790);

13   (Diet* adj3 treatment*).tw. (19,800);

14   (Diet* adj3 factor*).tw. (15,625);

15   ((Nutrient* or nutrition*) adj3 therap*).tw. (7105);

16   ((Nutrient* or nutrition*) adj3 support*).tw. (13,846);

17   ((Nutrient* or nutrition*) adj3 treatment*).tw. (4639);

18   Vitamin D.mp. [mp = title, abstract, original title, name of substance word, subject heading word, floating sub-heading word, keyword heading word, organism supplementary concept word, protocol supplementary concept word, rare disease supplementary concept word, unique identifier, synonyms] (77,196);

19   Vitamin E.mp. [mp = title, abstract, original title, name of substance word, subject heading word, floating sub-heading word, keyword heading word, organism supplementary concept word, protocol supplementary concept word, rare disease supplementary concept word, unique identifier, synonyms] (40,155);

20   (Curcumin or turmeric).tw. (17,317);

21   Omega-3.tw. (16,287);

22   PUFA*.mp. [mp = title, abstract, original title, name of substance word, subject heading word, floating sub-heading word, keyword heading word, organism supplementary concept word, protocol supplementary concept word, rare disease supplementary concept word, unique identifier, synonyms] (16,198);

23   DHA.mp. [mp = title, abstract, original title, name of substance word, subject heading word, floating sub-heading word, keyword heading word, organism supplementary concept word, protocol supplementary concept word, rare disease supplementary concept word, unique identifier, synonyms] (15,536);

24   EPA.mp. [mp = title, abstract, original title, name of substance word, subject heading word, floating sub-heading word, keyword heading word, organism supplementary concept word, protocol supplementary concept word, rare disease supplementary concept word, unique identifier, synonyms] (19,809);

25   Fish oil*.tw. (10,484);

26   (Calorie* adj3 restrict*).tw. (3820);

27   Glycaemic index*.tw. (2568);

28   Or/4-27 (520,887);

29   3 and 28 (754);

30   Limit 29 to (english language and yr = “2006–2021” and “humans only (removes records about animals)”) (540).

***************************

Database: Embase <1980 to 2021 Week 24>

Date: 23 June 2021

Search Strategy:

--------------------------------------------------------------------------------

1   Systemic lupus erythematosus/(91,844);

2   Systemic lupus erythematosus.mp. [mp = title, abstract, heading word, drug trade name, original title, device manufacturer, drug manufacturer, device trade name, keyword, floating subheading word, candidate term word] (102,178);

3   1 or 2 (102,178);

4   Dietary supplement/ (15,870);

5   Exp vitamin D/(146,946);

6   Alpha tocopherol/(68,164);

7   Curcumin/(27,880);

8   Exp omega-3 fatty acid/(33,919);

9   Diet/or healthy diet/or low-calorie diet/or low carbohydrate diet/or low glycemic index diet/or mediterranean diet/(231,760);

10   Low fat diet/(10,755);

11   Protein restriction/(7963);

12   Sodium restriction/(9312);

13   Dietary reference intake/(3492);

14   Nutritional status/(69,359);

15   Dietary supplements.mp. [mp = title, abstract, heading word, drug trade name, original title, device manufacturer, drug manufacturer, device trade name, keyword, floating subheading word, candidate term word] (12,216);

16   (Diet* adj3 intervention*).tw. (19,487);

17   (Diet* adj3 treatment*).tw. (23,247);

18   (Diet* adj3 factor*).tw. (19,314);

19   ((Nutrient* or nutrition*) adj3 therap*).tw. (10,525);

20   ((Nutrient* or nutrition*) adj3 support*).tw. (20,229);

21   ((Nutrient* or nutrition*) adj3 treatment*).tw. (6423);

22   Vitamin D.mp. [mp = title, abstract, heading word, drug trade name, original title, device manufacturer, drug manufacturer, device trade name, keyword, floating subheading word, candidate term word] (131,628);

23   Vitamin E.mp. [mp = title, abstract, heading word, drug trade name, original title, device manufacturer, drug manufacturer, device trade name, keyword, floating subheading word, candidate term word] (33,711);

24   (Curcumin or turmeric).tw. (22,516);

25   Omega-3.tw. (22,078);

26   PUFA*.mp. [mp = title, abstract, heading word, drug trade name, original title, device manufacturer, drug manufacturer, device trade name, keyword, floating subheading word, candidate term word] (20,532);

27   DHA.mp. [mp = title, abstract, heading word, drug trade name, original title, device manufacturer, drug manufacturer, device trade name, keyword, floating subheading word, candidate term word] (20,634);

28   EPA.mp. [mp = title, abstract, heading word, drug trade name, original title, device manufacturer, drug manufacturer, device trade name, keyword, floating subheading word, candidate term word] (23,366);

29   Fish oil*.tw. (13,253);

30   (Calorie* adj3 restrict*).tw. (5032);

31   Glycaemic index*.tw. (3612);

32   Or/4-31 (709,272);

33   3 and 32 (2630);

34   Limit 33 to (English language and yr = “2006–2021” and “humans only (removes records about animals)”) (2112).

***************************

Database: Cochrane Library

Date: 24 June 2021

ID Search

#1 MeSH descriptor: [Lupus Erythematosus, Systemic] this term only (844);

#2 (“systemic lupus erythematosus”) (2221);

#3 #1 OR #2 (2355);

#4 MeSH descriptor: [Dietary Supplements] this term only (10,860);

#5 MeSH descriptor: [vitamin D] explode all trees (5612);

#6 MeSH descriptor: [vitamin E] this term only (2122);

#7 MeSH descriptor: [Curcumin] this term only (431);

#8 MeSH descriptor: [Fatty Acids, Omega-3] explode all trees (3169);

#9 “Dietary supplement*” (2750);

#10  “Diet*” adj3 “intervention*” (415);

#11  “Diet*” adj3 “treatment*” (415);

#12  “Diet*” adj3 “factor*” (286);

#13  ((“Nutrient*” or “nutrition*”) adj3 “therap*”) (94);

#14  ((“Nutrient*” or “nutrition*”) adj3 “support*”) (417);

#15  ((“Nutrient*” or “nutrition*”) adj3 “treatment*”) (440);

#16  “Vitamin D” (13,267);

#17  “Vitamin E” (5064);

#18  “Curcumin” or “turmeric” (1544);

#19  “Omega-3” (6320);

#20  “PUFA*” (2042);

#21  “DHA” (3324);

#22  “EPA” (3314);

#23  “Fish oil*” (3034);

#24  “Calorie*” adj3 “restrict*” (20);

#25  “Glycaemic index*” (2350);

#26  #4 OR #5 OR #6 OR #7 OR #8 OR #9 OR #10 OR #11 OR #12 OR #13 OR #14 OR #15 OR #16 OR #17 OR #18 OR #19 OR #20 OR #21 OR #22 OR #23 OR #24 OR #25 (42,291)

#27  #3 AND #26 with Cochrane Library publication date Between Jan 2006 and Dec 2021 (129).

In the 129 records, 26 belong to Cochrane Reviews, 1 belongs to Cochrane Protocols, 102 are clinical trials.
